# A multilayer-multiplexer network processing scheme based on the dendritic integration in a single neuron

**DOI:** 10.3934/Neuroscience.2022006

**Published:** 2022-02-28

**Authors:** Jhunlyn Lorenzo, Stéphane Binczak, Sabir Jacquir

**Affiliations:** 1 Laboratoire ImViA EA7535, Université de Bourgogne, 9 Avenue Alain Savary, 21078 Dijon, France; 2 College of Engineering and Information Technology, Cavite State University, Indang, 4122, Philippines; 3 Université Paris-Saclay, CNRS, Institut des Neurosciences Paris-Saclay, 91400, Saclay, France

**Keywords:** CA3 neuron, computational model, dendritic integration, dendritic nonlinearity, dynamic threshold, input-output transformation, neuron model

## Abstract

Advances in neuronal studies suggest that a single neuron can perform integration functions previously associated only with neuronal networks. Here, we proposed a dendritic abstraction employing a dynamic thresholding function that models the spatiotemporal dendritic integration process of a CA3 pyramidal neuron. First, we developed an input-output quantification process that considers the natural neuronal response and the full range of dendritic dynamics. We analyzed the IO curves and demonstrated that dendritic integration is branch-specific and dynamic rather than the commonly employed static nonlinearity. Second, we completed the integration model by creating a dendritic abstraction incorporating the spatiotemporal characteristics of the dendrites. Furthermore, we predicted the dendritic activity in each dendritic layer and the corresponding somatic firing activity by employing the dendritic abstraction in a multilayer-multiplexer information processing scheme comparable to a neuronal network. The subthreshold activity influences the suprathreshold regions via its dynamic threshold, a parameter that is dependent not only on the driving force but also on the number of activated synapses along the dendritic branch. An individual dendritic branch performs multiple integration modes by shifting from supralinear to linear then to sublinear. The abstraction includes synaptic input location-dependent voltage delay and decay, time-dependent linear summation, and dynamic thresholding function. The proposed dendritic abstraction can be used to create multilayer-multiplexer neurons that consider the spatiotemporal properties of the dendrites and with greater computational capacity than the conventional schemes.

## 1. Introduction

Synaptic inputs entering the dendritic heads diffuse along the dendritic shaft and pass through a series of attenuation, amplification, filtering, and delay depending on the passive and active mechanisms in the dendritic compartments. Identifying the influence of the dendrites in a single input signal is a rudimentary process. However, the driving force of a single synaptic input is inadequate to influence a somatic spike. Therefore, multiple presynaptic cells continuously bombard the dendrites with synaptic inputs, and this is where the dendritic computation gets complicated. During simultaneous synaptic activation, the driving force of an input signal combines with the driving forces of its neighboring synaptic inputs. The contribution of a signal depends on several factors: its distance from the points of entry of other inputs, the diameter and length of its dendritic compartment, the morphology of the dendrites, the distribution of active channels, and the somatic dynamics, as well [1]–[5]. If the sum of the driving forces reaching the somatic compartment is above the spiking threshold, an action potential (AP) is generated. At this point, it is challenging to determine the influence of synaptic input on the somatic depolarization or to identify the role of the dendrites in neuronal computation [5]–[7]. Even after decades of studies, developing a unified understanding of how dendrites integrate and transform synaptic inputs entering the dendritic arborization into information-carrying spiking patterns is still lacking.

The pioneering studies of Rall in the 1960s [8], [9] paved the way for extensive research to determine the governing principles of dendritic integration [10], the crucial roles of dendrites in neuronal processing [11], and to create models or abstractions for improving the computational capacity of a neuron [12]. The biologically-inspired McCulloch-Pitts neuron model describes the dendritic integration process in its simplest abstraction [13]. The linear summation of weighted synaptic inputs passes through a nonlinear thresholding function that determines the spiking behavior of the neuron. The most recent dendritic abstraction is an extension of the linear-nonlinear Poisson (LNP) called the generalized linear model (GLM). In GLMs, the convolution of inputs passes through a static nonlinearity and is then fed to a spiking mechanism for the instantaneous firing rate [4], [14]. For both abstractions, the thresholding function (which is also called activation or transfer function) in the McCulloch-Pitts model and the static nonlinearity in GLM serves as the quantified function for dendritic integration. Most dendritic abstractions suggest that the nonlinearity is a sigmoidal signal [15]–[18], although it becomes increasingly implausible when fitted with biological data [19]. For inputs within the same branch of the pyramidal neurons, the subthreshold nonlinearity consists of three divisions: linear for weak signals, supralinear for intermediate signals, and sublinear for strong inputs [20]. Sigmoidal nonlinearity then becomes inaccurate when the dendritic parameters and input distribution change [21].

A widely used method for quantifying dendritic integration is the input-output (IO) transformation, comparing the synaptic inputs and the corresponding response in the somatic depolarization [16], [21], [22]. However, the IO curves vary significantly depending on which parameter is under observation, such as dendritic morphology, synaptic topology, and properties, ion channels, or combinations. Therefore, the level of biological realism or the complexity of the neuron model used for investigation is crucial for determining the dendritic nonlinearity [11]. Another factor to be considered in quantifying the dendritic integration is how the synaptic inputs were injected. Stimulation protocols, in modeling and physiological experiment, commonly use unitary or paired-pulse inputs [21], [23], [24]. However, such inputs limit the dynamic range of the dendrites. Because experimental recording and dendritic manipulation are challenging [11], the stimulation protocols and experiments on dendritic integration performed under simplified conditions *in vitro* or unobserved inputs *in vivo* do not conclusively describe the biophysical dynamics of the neuron [16]. Singh and Zald [25] introduced a static linear hook transfer function describing linear-nonlinear dendritic integration by performing IO transformation with the neuron model. However, the formulation of the transfer function sacrificed some dendritic properties due to the simplification of the dendritic morphology, removal of dendritic mechanisms, and application of a time-invariant function. Simplification or tuning of such properties has the potential to introduce some bias in the output [26].

Our goal here is two-fold: (1) formulate a thresholding function that captures the linear-nonlinear dendritic integration, both in the sub- and suprathreshold region, and (2) propose a dendritic abstraction that takes into account the spatiotemporal synaptic and dendritic dynamics. First, we created a CA3 pyramidal neuron model, which included most biophysical mechanisms distributed somatodendritically. Then, we simulated the model using *in vivo*-like synaptic inputs to reconstruct biophysical dynamics. We identified the synaptic input propagation along the dendritic length and formulated the corresponding model for the signal delay and attenuation. Next, we proposed a method for IO quantification for continuous inputs. Based on the results of the IO-transformation, we formulated the dynamic nonlinear thresholding functions. We described the functional components of the dendritic abstraction, which considers the spatial specificity of the inputs and the interaction between the inputs and dendritic mechanisms. Using multiple regression analysis, we identified the thresholding function. The resulting threshold nonlinearity is a dynamic function dependent on both the synaptic input amplitude and the number of activated synapses capturing the linear, supralinear, and sublinear dendritic integration processes in the subthreshold region. The proposed dendritic abstraction captures the spatiotemporal processes such as synaptic input attenuation and delay, synaptic-input location dependency of somatic depolarization, and the biophysical spiking mechanism of the neuron.

The next section, Section 2, presents the step-by-step procedure for formulating the thresholding function and the dendritic abstraction. In Section 3, we present the resulting model and describe its properties. Lastly, we have Section 4 for further discussion.

## 2. Methods

### 2.1. Pyramidal Neuron Model

A large number of time-dependent synaptic inputs entering a morphologically complex dendritic tree is difficult to control, and analyzing the dynamic response of the neuron is quite challenging [2], [27]. Thin oblique dendrites protruding from the main dendritic branch also influence the somatic response. Therefore, the neuron model should be biophysically-plausible, with its morphology and distributed mechanisms, but simple enough in order to follow the signal propagation.

#### 2.1.1. Distributed Mechanisms

**Figure 1.**
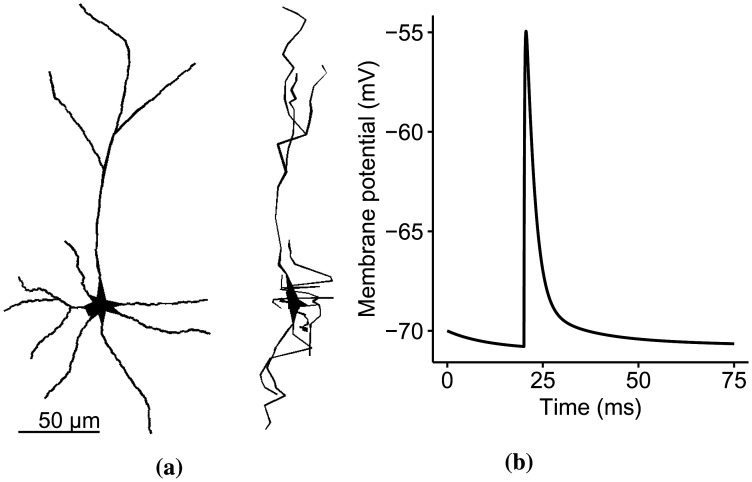
(a) The reconstructed model of the CA3 pyramidal neuron. The morphology of the pyramidal neuron in XY- and YZ axes (left and right, respectively) displays anisotropic features. (b) A single synaptic input. The single pulse synaptic input activated at *t* = 20 ms and with a maximum amplitude of −55.19 mV is a combination of AMPARs and NMDARs kinetics and follows a double-exponential rise and decay function.

We developed a simple but biologically-plausible anisotropic model of the CA3 pyramidal neuron from a rat hippocampus [28] using the NEURON simulation platform [29] ran with a time step of 0.05 ms [27]. The morphological detail is available at NeuroMorpho.Org, with an ID number NMO_76005 [30]. This simple model has seven stems, four bifurcations, 15 dendritic branches, and a total of 116 dendritic sections. The spines are mushroom-typed with a head diameter of 0.35 *µ*m [28], a neck diameter of 0.1 *µ*m [31], and a neck length of 0.35 *µ*m [28]. The spines are placed 0.5 *µ*m from one another along the dendritic length [28], equating to 2129 synaptic locations. The d_lambda discretization rule divided the dendritic sections into electrical compartments [32]. Figure 1a illustrates the anisotropic and morphologically realistic model.

The neuron dynamics consist of various interactions amongst biophysical mechanisms such as synaptic saturations, dendritic spikes, and N-methyl-D-aspartate (NMDA) receptor nonlinearities [16], [24], [33]. These mechanisms facilitate dendritic integration and control the transformation of synaptic input patterns into somatic membrane potential changes and the generation of output spike trains [4], [16]. Therefore, we incorporate passive mechanisms and active channels into the neuron. Table 1 presents the properties of these mechanisms and their distribution throughout the somatodendritic arborization. The neuron has constant passive properties, which include the membrane and cytoplasmic resistivities and specific capacitance. In the spine necks, the cytoplasmic resistivity was set so that the spine neck resistance is equal to 500 MΩ [31]. Furthermore, the biological cell model includes active channels distributed heterogeneously from soma to dendrites. These active channels are the fast activating sodium channels (Na), delayed-rectifier potassium channels (K_DR_), A-type potassium channels (K_A_), N-, T-, and L-type calcium channels (Ca_N_, Ca_T_, and Ca_L_).

**Table 1.** CA3 pyramidal neuron model distributed mechanisms.

Parameter	Value	Placement
Specific membrane capacitance *C_m_*	1 *µ*F/cm	soma, dendrites, spines [34]
Cytoplasmic resistivity	100 Ωcm	soma, dendrites, spine heads [34]
*R_a_*	1122 Ωcm	spine necks [34]
Leak conductance density *g_L_*	0.0001 S/cm^2^	soma, dendrites [35]
Fast Na^+^ conductance density *g_Na_*	0.03 S/cm^2^	soma, dendrites [35]
Delayed rectifier K^+^ conductance density ${\bar{g}}_{K_{DR}}$	0.015 S/cm^2^	soma, dendrites [35]
A-type K^+^ conductance	0.005 S/cm^2^	soma [35]
density *g_KA_*	${\bar{g}}_{K_{A_{soma}}}(1+5.2x/350)$	somatodendritic gradient where *x* is the distance from the soma in *µ*m [36]
N-type Ca^2+^ conductance density ${\bar{g}}_{Ca_{N}}$	0.0015 S/cm^2^	soma, spine heads [37], [38]
T-type Ca^2+^ conductance density ${\bar{g}}_{Ca_{T}}$	0.001 S/cm^2^	soma, dendrites [37]–[39]
L-type Ca^2+^ conductance density ${\bar{g}}_{Ca_{L}}$	0.0013 S/cm^2^	soma, spine heads, dendritic length of 50 *µ*m from the soma [37]–[39]

#### 2.1.2. Synaptic Inputs

In this study, the synaptic inputs follow *in vivo*-like spatiotemporal patterns to replicate the full range of neuronal dynamics covering both the sub- and suprathreshold regions. We modeled the spatial- and time-dependent excitatory synaptic inputs as membrane potentials induced by the conductance change of *α*-amino-3-hydroxy-5-methyl-4-isoxazole-propionic acid (AMPA) and NMDA receptors in the spine heads [40].

For time *t*, the synaptic conductance of AMPAr, *g_AMPA_*, is a double-exponential function [41] given by

$$g_{AMPA}(t)=g_{Amax}\alpha_{A}\left[e^{\left(-t/\tau_{A2}\right)}-e^{\left(-t/\tau_{A1}\right)}\right],$$ (1)

with peak synaptic conductance, *g_Amax_*, and rise and decay time constants, *τ_A_*_1_ and *τ_A_*_2_, respectively [36]. The NEURON Exp2Syn function models the AMPAR response [4]. The voltage-dependent NMDARs kinetics follow the same function as the AMPARs kinetics with extracellular Mg^2+^ blocking component [36], [37], [40]. The NMDAR conductance *g_NMDA_* is

$$g_{NMDA}(t)=g_{Nmax}\frac{\alpha_{N}\left[e^{\left(-t/\tau_{N2}\right)}-e^{\left(-t/\tau_{N1}\right)}\right]}{1+\frac{[Mg^{2+}]e^{-0.062V_{i}(t)}}{3.57}},$$ (2)

where *g_Nmax_* is the peak synaptic conductance, *τ_N_*_1_ and *τ_N_*_2_ are the rise and decay time constants, [*Mg*^2+^] is the extracellular concentration of Mg^2+^, and *V_i_* is the synaptic membrane potential [36]. The NEURON function automatically computes the values of *α_A_* and *α_N_* so that the maximum values of *g_AMPA_* and *g_NMDA_* are equal to their corresponding peak conductances. The single synaptic input in Figure 1b measured in the spine head has a 14.81 mV peak amplitude from rest and a half-width of 2.75 ms. The corresponding values of the synaptic input parameters, and other neuron properties are in Table 2. For a full CA3 model description, the NEURON code is available in the supplementary materials.

**Table 2.** Synaptic inputs and miscellaneous parameters.

Parameter	Value	Source
*Synaptic Inputs*		
AMPAR time rise *τ*_A1_	0.2 ms	[40], [41]
AMPAR time decay *τ*_A2_	2 ms	[40]
AMPAR peak conductance *g_Amax_*	0.5 nS	[36]
AMPAR reversal potential *E_AMPA_*	0 mV	[41]
NMDAR time rise *τ_N_*_1_	2 ms	[40]
NMDAR time decay *τ_N_*_2_	86 ms	[40]
NMDAR peak conductance *g_Nmax_*	0.16 nS	[36]
NMDAR reversal potential *E_NMDA_*	-5 mV	[42], [43]
Extracellular [*Mg*^2+^]_*o*_	1 mM	[36], [44]
*Miscellaneous*		
Resting potential *V_R_*	-70 mV	[40]
Leak reversal potential *E_L_*	-70 mV	[40]
Na^+^ reversal potential *E_Na_*	50 mV	[45]
K^+^ reversal potential *E_K_*	-90 mV	[45]
Intracellular [*Ca*^2+^]_*i*_	50×10^−6^ mM	[45]
Extracellular [*Ca*^2+^]_*o*_	2 mM	[45]

### 2.2. Dendritic Abstraction

**Figure 2.**
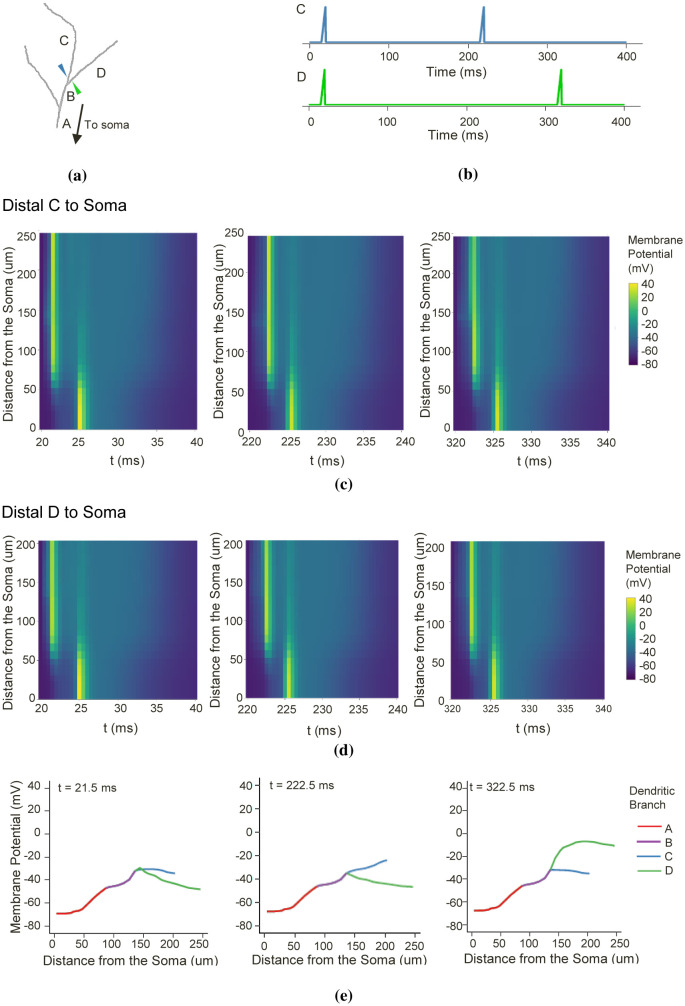
Signal propagation along the apical dendritic branches. (a) At a distance of 2 *µ*m from the branching point, ten excitatory inputs activate clustered synapses along dendritic branches C and D with their respective activation patterns in (b). The space plots in (c) and (d) show the change in membrane potential of the dendritic length from the distal end of C to soma and from the distal end of D to soma, respectively. (e) Further investigation on the membrane potential during synaptic activation indicates that even though the signals diffuse in all directions, it is still possible to identify the dendritic branch that drives the somatic depolarization by determining the synaptic locations that is first to peak.

When multiple synapses are simultaneously active and spatially segregated, inputs driving the somatic potential change are challenging to discriminate. Working on the notion that dendrites require dynamic independence to perform various computations, we identified each dendritic length as an independent subunit. For example, when identical inputs simultaneously activate clustered synapses in C and D (Figure 2a) following spiking patterns in Figure 2b, the changes in the membrane potential along the distal C-to-soma (Figure 2c) and distal D-to-soma (Figure 2d) at specific time windows indicate signal diffusion. Synaptic inputs propagate from the synaptic location to the distal end of the dendritic branch, to the neighboring branches and the soma. Indeed, signal propagation along the dendritic branch is bi-directional, and signals backpropagate, upon AP generation, from the soma to some extent of the distal branches [46]. This bidirectional propagation then makes input discrimination intricate. Notice the low membrane potential between the synaptic input activation and the somatic activation. It suggests that even though the synaptic input in the tertiary dendrites significantly attenuates, it can still cause a somatic activation with ~3 ms delay. Moreover, the backpropagating AP may have a minimum effect on the distal dendrites. We examined the membrane potential magnitudes, particularly during the synaptic input peaks (Figure 2e). At *t* = 21.50 ms, both the dendrites drive the membrane potential elevation. When individual dendrite is active, at *t* = 222.50 ms and *t* = 322.50 ms, significant differences in the membrane potential between sibling branches are noticeable. Therefore, even though signal diffuses throughout the dendritic tree, we can still identify the origin of the somatic fluctuations by the level of the driving force produced from the individual branches. We repeated the simulations using varying synaptic input combinations and locations, and the results were similar. One of the foci of this study is to identify how the dendrites process synaptic inputs to perform branch dendrite-specific computation. With these results, we, therefore, arrive with the following modeling approach.

We already presented the biological model of the neuron in Subsection 2.1. The next step is to identify a dendritic abstraction that models the spatiotemporal dendritic integration process. This abstraction is necessary because the thresholding nonlinearity, a time-independent function, is not sufficient for describing the dendritic dynamics [16], [25]. There is a need for another function for the signal propagation along the dendritic branch, the corresponding signal delay, attenuation, and the time- and location-dependency.

#### 2.2.1. Model

**Figure 3.**
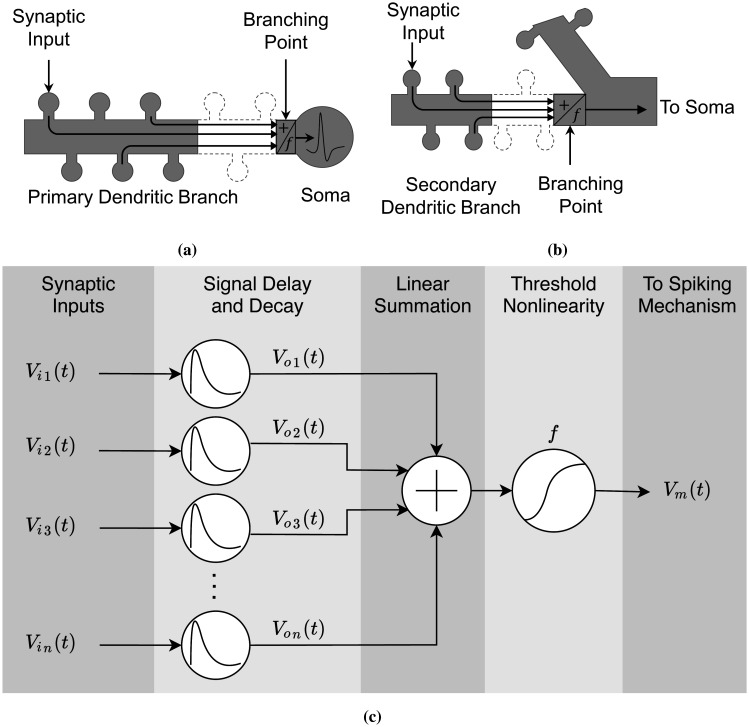
The dendritic branch is an independent computational subunit. The synaptic inputs enter the dendritic shaft via the dendritic neck and then flow untangled in one direction, from the distal end of the branch to the opposite end. (a) The primary dendritic branch is from the first bifurcation point to the section connected to the soma, while (b) the secondary dendritic branch is from the distal end to the bifurcation point. Dendritic integration and transformation occur in the branching point. Its output drives the somatic spiking in (a) or travels to the soma in (b). (c) The equivalent dendritic abstraction includes the time-continuous synaptic inputs *V_in_*(*t*), that experience individual attenuation and delay. The outputs *V_on_*(*t*) are linearly integrated and then sent to the thresholding function for transformation. The transformed signal *V_m_*(*t*) flows to the next proximal point until it reaches the somatic compartment for AP generation.

Consider the primary dendritic branch (the apical trunk) and the secondary dendritic branch (in the apical tuft) shown in Figure 3a and Figure 3b. For modeling purposes, we apply the following concepts:

Each dendritic branch is an independent computational subunit. The dendritic abstraction corresponds to the dynamics occurring within a compartment: the dendritic length from the bifurcation point to the somatic connection (Figure 3a) or from the distal end of the apical dendritic branch to the bifurcation point (Figure 3b). Based on the impedance tree-graph concept [47], branches are mutually independent.
Synaptic activation occurs in the dendritic spine head and none along the dendritic shaft. This assumption is to avoid electrical shunting and over saturation of inputs [31] and ensure that the synaptic inputs have a comparable influence on the dendritic shaft around their entry points. Therefore, we focus on integrating excitatory synaptic inputs on the dendritic heads without any inhibitory inputs entering the dendritic shaft. Inhibitory signals have shunting and subtractive effects that may prevent somatic spiking [48].
Signals within the dendritic branch propagate individually (untangled), and synaptic inputs travel only in one direction, from the distal apical or basal dendrites to the somatic compartment [47]. We considered the dendritic branch as a multiplexer, and the signals are superimposed on each other. It simplifies the tangling problem and allows us to approximate the influence of each input to the depolarization in the branching point.
The input summation and IO transformation occur in the branching point. Theoretically and experimentally, and when we consider the equivalent dendritic circuit, a synaptic input almost immediately integrates with the neighboring inputs around its entry point in the dendritic shaft. Therefore, dendritic integration can transpire anywhere along the dendritic length. To cover the whole dendritic length and the incoming signals, we take the proximal end of the dendritic branch as the thresholding point.

The dendritic abstraction consists of the following. The synaptic input *V_in_*(*t*), where *n* is the number of the activated synapse, is a time-varying *in vivo*-like synaptic spiking. The dendritic active and passive mechanisms, throughout the distance between the dendritic head to the branching point, influence the amplitude decay and time delay of *V_in_*(*t*). The attenuation function output *V_on_*(*t*) is a weaker location-dependent signal. The linear summation integrates the attenuated signals and thus represents the linear component of dendritic integration. The nonlinear function *f* transforms the sum into a single information-carrying signal *V_m_*(*t*). *V_m_*(*t*) then travels along the dendritic arborization to the next proximal dendritic branch or straight to the soma for spiking.

#### 2.2.2. Signal propagation and delay

Wybo et al. [49] presented a method for simplifying the dendritic morphology directly from experimental data and suggested that a complex subtree can be modeled into a single branch with multiple reduced compartments while preserving the neuronal biological responses. They approximated the dendritic voltage responses using the Hodgkin-Huxley formalism, where the parameters for the passive and active channels were fitted using the least-square method. Since our goal is to identify the input integration at the thresholding point, dividing the dendritic branch into compartments with multiple nonlinear systems of equations is computationally costly. In order to simplify the dendritic voltage response, we used the voltage propagation model, which was modified to incorporate the collective effects of the passive and active mechanism along the cable of varying morphology. Instead of fitting each ion channel in the compartment, we identified the parameters *α* and *β* in Equation 3 using regression analysis of the membrane potential at the entry and thresholding points caused by individual synaptic inputs.

The IO transformation method usually compares the arithmetic summation of input with the output depolarization. However, this method disregards the synaptic locations and considers that the synaptic inputs have the same influence over the soma or the thresholding point. Synaptic input travels through the spine neck from the spine head, enters the dendritic shaft, and then propagates up to the thresholding point. While propagating, signals are susceptible to considerable voltage decay and delay before reaching the proximal end of the dendritic branch. Therefore, it is only fitting to compute the arithmetic sum of the decayed and delayed signals rather than the direct sum of the signals entering the spine head. In this case, the dendritic abstraction becomes spatiotemporal, where the input signals are subjected to decay and delay by *V_o_*(*t*) before integrating into the thresholding point (Figure 3c). The spatiotemporal propagation model is defined as

$$V_{o}(t)=\alpha e^{-x/\lambda }V_{i}\left(t-\beta \frac{\tau x}{2\pi }\right),$$ (3)

where *V_o_* is the attenuated and delayed synaptic input amplitude when it reaches the thresholding point at time *t*, *V_i_* is the electrotonic potential of the synaptic input (the difference between the synaptic input and the resting potential), and *x* is the distance between the synaptic input entry point and the thresholding point [50]–[52]. The propagation velocity varies at different points along the dendritic branch due to the difference in diameters and lengths of the dendritic sections. To simplify this issue, we converted the dendritic branch into a single cable with constant distributed parameters by computing for the total effective length, *λ*, and total time constant, *τ* [52]. The total effective length (in *µ*m), given as

$$\lambda =\sum_{j=1}^{k}\sqrt{\frac{R_{mj}}{R_{aj}}\frac{d_{j}}{4}},$$ (4)

and the total time constant (in ms), given as

$$\tau =\sum_{j=1}^{k}R_{mj}C_{mj},$$ (5)

are dependent on the diameter *d* of *k* sections. Here, *R_m_* is the membrane resistance equal to 10 000 Ωcm^2^, and *R_a_* is the axial resistance equal to 100 Ωcm [50], [52], consistent with the biological model. The parameter *α* captures the dramatic decrease in synaptic input amplitude as it travels along the high resistance spine neck [34] and dendritic shaft, while *β* defines the signal delay caused by the active mechanisms along the dendritic length. The terms *αe*^(−*x*/*λ*)^ is the coefficient of voltage decay and *β*(*τx*/2*π*) represents the time delay. We computed the corresponding values of *α* and *β* for each dendritic branch via regression analysis. To do so, each synapse along the dendritic branch, from the distal to the proximal end, is activated one at a time while measuring the membrane potentials at the input spine head and the thresholding point. The parameter *α* was computed by fitting *αe*^−*x*/*λ*^ to the amount of measured voltage decays (difference between the peak depolarizations of the synaptic inputs and thresholding point). In parallel, *β* was determined by fitting *β*(*τx*/2*π*) to the measured voltage delays (the time difference between the peak depolarizations at the spine head and thresholding point).

#### 2.2.3. Spiking mechanism

Even with the ramified morphology of dendrites and spatiotemporal disparity of synaptic inputs, it is the soma that is responsible for encoding information [6]. When the transformed dendritic signals arriving at the soma are large enough, the somatic spiking mechanism generates an AP [21]. In the dendritic abstraction, we used the Hodgkin-Huxley spiking mechanism in Equation 6 because it closely reproduces the somatic dynamics of the biological neuron.

$$C\frac{dV}{dt}=I-g_{Na}m^{3}h\left(V-V_{Na}\right)-g_{K}n^{4}\left(V-V_{K}\right)-g_{Ca}s^{2}r\left(V-V_{Ca}\right)-g_{L}\left(V-V_{L}\right)$$ (6)

It includes active Na^+^, *K*^+^, and Ca^2+^ channels and passive leak parameters. *V* corresponds to the membrane potential, *C* is the membrane capacitance, and *I* serves as the input whose intensity is equal to 10 *µ*A/cm^2^ multiplied by the summation of activation functions *f* of the dendritic branches connected to the soma [53]–[56]. Equation 7 gives the channel activation and inactivation functions. Refer to Table 3 for the parameters.

$$\frac{dz}{dt}=\alpha_{z}(1-z)-\beta_{z}z,\;z=m,n,h,r,s,$$ (7)

$$\begin{array}{ll} \alpha_{m}=\frac{0.1\left(-V-45\right)}{\text{exp}\left(\frac{-V-45}{10}\right)-1}, & \beta_{m}=4\text{exp}\left(\frac{-V-70}{18}\right),\\ \alpha_{n}=\frac{0.01\left(-V-60\right)}{\text{exp}\left(\frac{-V-60}{10}\right)-1}, & \beta_{n}=0.125\text{exp}\left(\frac{-V-70}{80}\right),\\ \alpha_{h}=0.07\text{exp}\left(\frac{-V-70}{30}\right), & \beta_{h}=\frac{1}{\text{exp}\left(\frac{-V-40}{10}\right)+1},\\ \alpha_{r}=0.000457\text{exp}\left(\frac{-V-13}{50}\right), & \beta_{r}=\frac{0.0065}{\text{exp}\left(\frac{-V-15}{38}\right)+1},\\ \alpha_{s}=\frac{0.055\left(-V-27\right)}{\text{exp}\left(\frac{-V-37}{3.8}\right)-1}, & \beta_{s}=0.094\text{exp}\left(\frac{-V-75}{17}\right). \end{array}$$

**Table 3.** Somatic spiking mechanism.

Parameter	Value	Description
*g_Na_*	120	Na^+^ conductance (mS/cm^2^)
*g_K_*	36	K^+^ conductance (mS/cm^2^)
*g_Ca_*	7	Ca^2+^ conductance (mS/cm^2^)
*g_L_*	0.1	Leak conductance (mS/cm^2^)
*V_Na_*	45	Na^+^ reversal potential (mV)
*V_K_*	-75	K^+^ reversal potential (mV)
*V_Ca_*	90	Ca^2+^ reversal potential (mV)
*V_L_*	-70	Leak reversal potential (mV)
*C*	1	Membrane capacitance (*µ*F/cm^2^)

For clarification, the dendritic abstraction can employ other spiking mechanisms such as leaky-integrate and fire (LIF). In this case, we employ the common Hodgkin-Huxley formulism, which best approximates the spiking behavior and the shape of the AP of the biological soma consisting of multiple types of ionic channels presented in Subsection 2.1.

### 2.3. Input-Output Transformation

There are some issues we need to address before proceeding with the IO transformation process. First, commonly employed stimulation protocols are the single-pulse or paired-pulse stimulation. These inputs are simple and do not replicate the dynamics and sustained *in vivo* inputs [16]. The simplified experimental conditions do not entirely capture the dynamic behavior of the neuron and thus results in incoherent concepts between *in vitro* and *in vivo* integration process [57]. Second, experiments and modeling procedures block the spiking mechanisms of the neuron to avoid AP generation and backpropagation [16]. By doing so, the dynamics remain in the subthreshold region. Lastly, IO quantification protocols directly compare the input summation with the somatic potential [24]. It disregards the influence of the nonlinear mechanisms along the dendrites and the spatiotemporal features of the inputs.

In this subsection, we present a method for quantifying dendritic integration addressing the issues above. The synaptic inputs are *in vivo*-like with time-varying spiking patterns and stochastic noise. We also keep the natural response of the soma and record a more extensive dynamic range, from sub- to suprathreshold. Then, the IO quantification compares *V_expected_*, the expected depolarization (the sum of inputs reaching the thresholding point), and *V_observed_*, the observed depolarization (the measured depolarization at the thresholding point). By quantifying the IO relationship from a single point, we retain the spatiotemporal characteristics of the signals.

#### 2.3.1. Input distribution

For each dendritic branch, we performed 40 simulations that lasted 1000 ms each (with a 0.05 ms time step) and whose inputs have a Poisson spiking interval of 20 ms and Gaussian synaptic noise. These inputs were either segregated or clustered along the dendritic branch. Let *d* be the length of the dendritic branch. For segregated inputs, activated synapses were randomly located between 0.01*d*–*d*, 0.01*d*–0.5*d*, or 0.5*d*–*d*. For clustered inputs, activated synapses were also randomly placed between 0.01*d*–0.25*d*, 0.25*d*–0.5*d*, 0.5*d*–0.75*d*, or 0.75*d*–*d*. The number of activated synapses per simulation was minimum enough to cause depolarization at the thresholding point and maximum enough to avoid oversaturation and excessive dendritic burst. Therefore, there were three, five, and seven synaptic inputs activated for each simulation set. In total, there were 21 input categories. There were ten simulations for segregated inputs along the whole dendritic branch and five simulations for the rest. This input distribution gave a wide range of synaptic and dendritic activities.

#### 2.3.2. Input-output quantification

**Figure 4.**
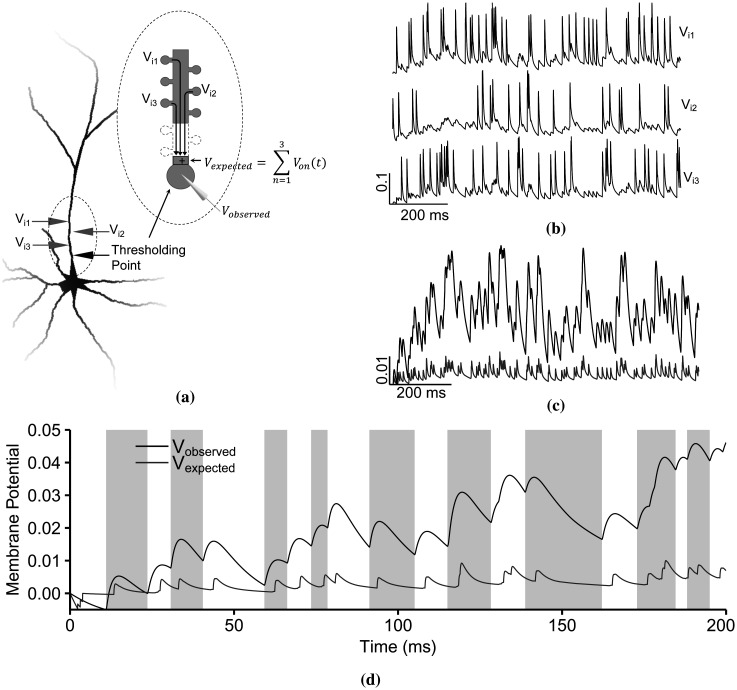
The input-output quantification process. (a) The *in vivo*-like synaptic inputs *V_i_*_1_, *V_i_*_2_, and *V_i_*_3_ were measured from the synaptic heads that are 46.67 *µ*m, 28.32 *µ*m, and 38.17 *u*m from the thresholding point of the primary apical dendritic branch. Inputs *V_in_* are subjected to propagation delay and decay before reaching the thresholding point for arithmetic summation, where the resulting sum is the *V_expected_*. The measured potential at the thresholding point is termed as *V_observed_*. (b) The random spiking frequency of the inputs results in a dynamic and sustained nature. (c) The summation of the predicted attenuated inputs reaching the thresholding point (gray) drove the subthreshold depolarization (black) from the same point. (d) Two consecutive local minima of the membrane potential (*V_observed_*) divided the observed and expected depolarization (*V_expected_*) into time windows (alternating white and gray rectangles). IO quantification is then the comparison between the maximum observed depolarization (black) and the maximum expected depolarization (gray) within the same time window.

The following are the features of this IO quantification framework: the input is the summation of the estimated synaptic inputs reaching the thresholding point (normalized), then the output is the measured membrane potential at the thresholding point (normalized), and lastly, the IO relationship is a nonlinear time-independent function. The feature scaling restricts the voltage amplitude from 0 (−70 mV) to 1 (40 mV) to avoid the effects of negative values. During the simulation, we measured the synaptic inputs at the spine head along the primary apical trunk as well as at the thresholding point (Figure 4a). Then, we estimated the voltage decay and delay of the individual inputs when they reached the thresholding point by applying Equation 3. *V_expected_* represents the summation of the attenuated inputs. For instance, Figure 4b shows the input patterns measured from synapses segregated along the primary apical dendritic branch. The input summation (gray) shown in Figure 4c drove the depolarization at the thresholding point (black).

For the IO quantification, first, we divided the *V_observed_* time series into time windows defined by the two consecutive local minima (gray lines in Figure 4d). The same time windows were also applied to *V_expected_*. Within each time window, we measured the maximum depolarization of *V_observed_* and the corresponding maximum peak amplitude of *V_expected_*. The comparison between these points gives us the IO relationship.

## 3. Results

### 3.1 Dendritic Integration in the Subthreshold and Suprathreshold Regions

The dendritic arborization in Figure 1a has three levels mainly, the primary dendrites (those connected directly to the soma), the secondary dendrites (after the first bifurcation points), and the tertiary dendrites (after the last bifurcation points). We simulated the neuron model for each dendritic branch by randomly activating segregated or clustered synaptic inputs along the dendritic length. Then, we applied the IO quantification method to identify the within-branch dendritic integration in the sub- and suprathreshold regions. Furthermore, we removed the suprathreshold IO pairs caused by the backpropagating signals and dendritic bursts from the dataset.

**Figure 5.**
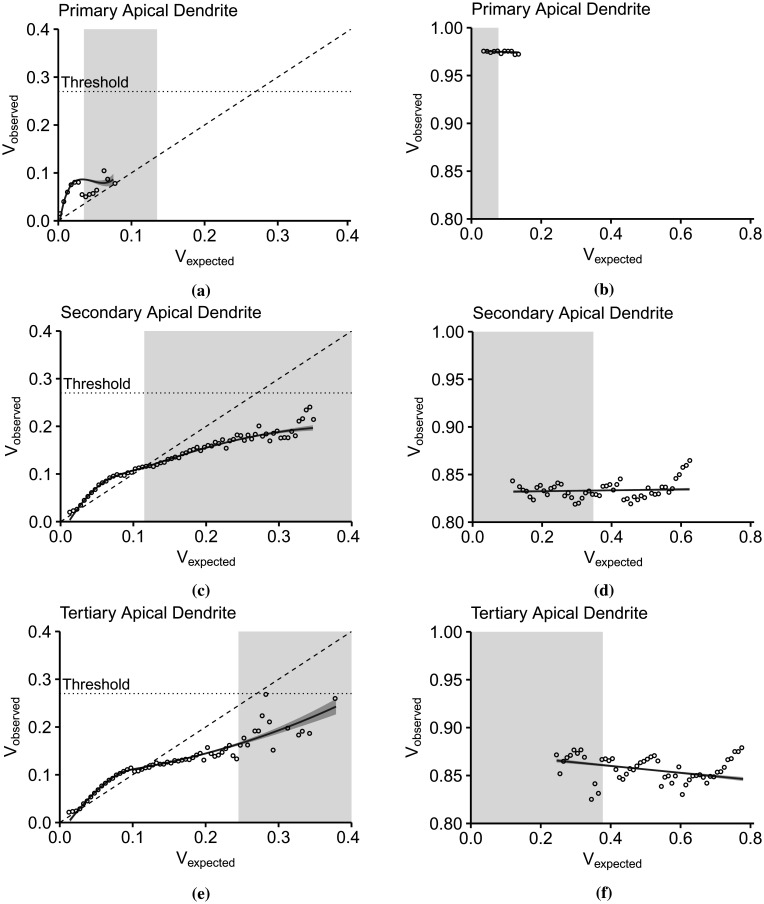
Dendritic integration in the subthreshold (left column) and suprathreshold (right column) regions. (a) The subthreshold IO curve in the primary dendrite (dendritic branch connected to the soma) is strongly supralinear between [0, 0.1] of *V_expected_* and *V_observed_*, while in (b) the suprathreshold region, the spiking amplitude is constant at 0.975. (c) The secondary apical dendrite displays a nonlinearity that deviates from linear to supralinear, and after crossing *V_expected_* = 0.1 shifts to sublinear. (d) The range of *V_expected_* necessary for a successful spike exceeds that of (b) but with lower spiking amplitude (0.83). (e) The tertiary apical dendrite produces a subthreshold nonlinearity that is almost similar to (c), while the suprathreshold spiking amplitudes slopes down (*m* = −3.77). Moreover, the gray regions in the subthreshold plots (a, c, and e) represent the range of *V_expected_* causing a successful spike in the suprathreshold region, while the gray regions in the subthreshold plots (b,d, and f) indicates the range of subthreshold points. These boundaries indicate that the spiking threshold for each dendritic branch increases (0.035 in (a), 0.115 in (c), and 0.245 in (e)) with respect to the distance from the soma and the location in the arborization of the dendritic branch. Besides, the subthreshold and suprathreshold regions overlap with each other, suggesting that the thresholding function is not a simple sigmoid function.

Here, we present the IO curves in the apical trunk (primary), the left-most apical tuft (secondary), and the right-most apical tuft (tertiary) dendrites (Figure 1a). The subthreshold IO data were smoothened using the locally estimated scatterplot smoothing (loess) method, while linear models best described the suprathreshold data. The points represent the mean *V_observed_* amplitude per *V_expected_* bin width. Figure 5 illustrates the *V_expected_* : *V_observed_* curves of the apical dendrites in the subthreshold (left column) and the suprathreshold (right column) regions. According to [20], the subthreshold nonlinearity in the pyramidal neurons follows a linear-supralinear-sublinear curve based on increasing input strength. This relationship is visible in the secondary (Figure 5c) and tertiary dendrites (Figure 5e), where the IO curves deviate above and below the linearity upon crossing *V_expected_* = 0.1. However, the primary dendrite exhibits a strong supralinear dendritic integration (Figure 5a), then the *V_observed_* levels stabilize as the curve approaches the sublinear region. The basal dendrites connected to the soma also exhibit almost the same nonlinearity. In the suprathreshold regions (Figure 5b, 5d, and 5f), the *V_observed_* level is steady even with increasing *V_expected_*. It is as expected since the somatic spiking peak amplitude is constant.

Another striking feature of the IO transformation is the level of *V_observed_* and the range of *V_expected_*. In the primary dendrite (Figure 5a), the suprathreshold dendritic integration output lies between 0 and 0.1 of *V_observed_*, which is also the same input summation range. *V_expected_* > 0.1 results in somatic spiking. In the secondary (Figure 5c) and tertiary dendrites (Figure 5e), the dendritic integration plateaus after crossing *V_expected_* = 0.1 at a level lower than the threshold (*V_observed_* = 0.27). The input summation range causing a subthreshold response increases, from the primary to the tertiary dendrites (the dendritic branch gets further away from the soma). The smaller range of *V_expected_* in the primary dendrite correlates with the large voltage decay of synaptic inputs reaching the branching point. The peak of observed depolarization in the suprathreshold region also decreases with respect to the distance and number of bifurcations from the soma. In primary dendrite (Figure 5b), successful spiking produces a peak depolarization of 0.975 (37.5 mV), while in the secondary dendrite (Figure 5d), the peak is constant around 0.83 (21.30 mV). *V_observed_* in the tertiary dendrite (Figure 5f) slopes down from 0.86 (24.6 mV) to 0.84 (22.4 mV). Furthermore, the range of input summation that can result in successful spikes gets wider. In Figure 5b, successful spikes are concentrated between 0.035 to 0.135. In Figure 5d and 5f, spikes are spread within [0.115, 0.625] and [0.245, 0.775], respectively.

Furthermore, the minimum input amplitude necessary for a successful spike changes with dendritic location. The gray areas in subthreshold plots (Figure 5a 5c, and 5e) indicate the ranges of *V_expected_* that resulted in somatic spiking, while in the suprathreshold regions (Figure 5b, 5d, and 5f), the gray areas indicate the subthreshold ranges. The minimum input summation for suprathreshold spiking shifts to the right, from 0.035 in the primary branch (Figure 5a), 0.115 in the secondary branch (Figure 5c), to 0.245 in the tertiary branch (Figure 5e). In the dendritic integration viewed within-branched, it appears that the spiking threshold differs as a consequence of heterogeneity in distributed active mechanisms along the dendrites. Then, considering that the dendrites received the same input combinations in terms of the number of activated synapses (three, five, and seven), the range of subthreshold values that the dendrites produced expands. Moreover, the subthreshold and suprathreshold areas overlap. There are subthreshold values under the region where successful spikes occurred. It suggests that the thresholding function is not a straightforward sigmoid because a sigmoid function considers the points within the overlapping regions as errors.

The within-branch integration results demonstrate that the thresholding function is asymmetric, in contrast with a sigmoidal function. The subthreshold integration is a linear-supralinear-sublinear function dependent on the branch location within the dendritic arbor and whose degree of integration is influenced by the active mechanisms and the strength of the driving forces of the synaptic inputs.

### 3.2. Location-Dependent Dynamics

We analyzed the effects of segregated and clustered synaptic input locations on the within-branched dendritic integration. The density plots in Figure 10 of the Supplementary Materials show the distribution of *V_expected_*/*V_observed_* for each synaptic location along the dendritic branch. The plots suggest that synaptic locations have an inconsiderable influence on dendritic depolarization, which is a direct consequence of the application of synaptic input decay and delay (Equation 3) preceding the IO quantification. Therefore, we consider the dendritic integration as independent from the synaptic input location, given that the propagation model in the dendritic abstraction (Figure 3c) already has the spatiotemporal data of the input signals.

**Figure 6.**
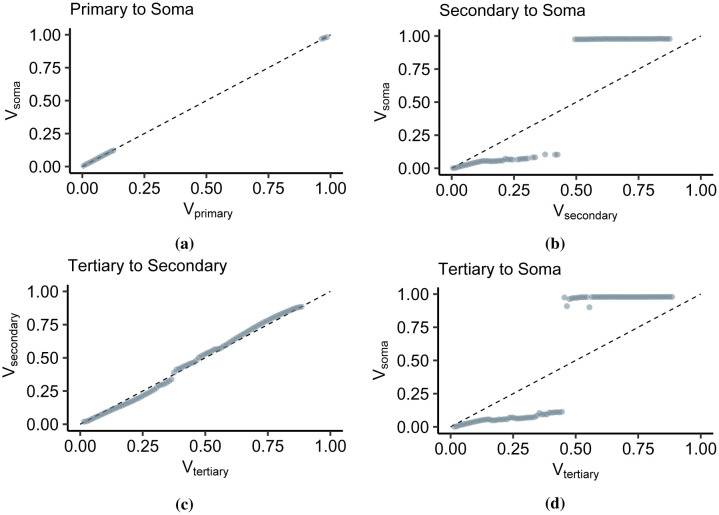
Location-dependent dynamics. The plots show the relationships between soma and dendritic branches. The points represent the mean output depolarization per input bins. (a). The depolarizations in the soma and the apical thresholding point form a linear relationship in the subthreshold (0 ≤ *V_primary_* ≤ 0.125) and concentrated in the suprathreshold with large discontinuity in between. (b) The relationship between *V_secondary_* and *V_soma_* is strongly sublinear in the subthreshold region (0 ≤ *V_secondary_* ≤ 0. 5) and constant with increasing *V_secondary_* inputs. (c) Overall, the relationship between *V_tertiary_* to *V_secondary_* is linear, with a slight fluctuation from the linearity. (c) The relationship between the tertiary branch inputs and the soma is comparable with that in (b) because the secondary thresholding point also transforms the inputs flowing from the tertiary dendrites.

Most studies in IO transformations view the observed depolarization at the soma and compare it to the summation of inputs or the intensifying stimulation from various dendritic sites. At this point, we compare the membrane potential at the thresholding point with the somatic depolarization (Figure 6). The primary apical dendrite is located near the soma; therefore, the membrane potential at the thresholding point (*V_primary_*) leads to an equivalent somatic potential (*V_soma_*) (Figure 6a). The subthreshold regions display a linear relationship while spike amplitudes rest at ~1. The discontinuity occurs due to the sudden increase in somatic depolarization and the proximity of the primary thresholding point to the soma. The primary dendrite has a low threshold in that the maximum subthreshold *V_primary_* is 0.125. The low threshold is consistent with the low *V_expected_* values in Figure 5a. However, the signal emanating from the secondary apical dendrite undergoes a substantial voltage decay (Figure 6b) due to the drastic increase in apical trunk resistance. The subthreshold region displays a sublinear relationship with the somatic depolarization. In this case, the first successful spike occurs at *V_secondary_* = 0.5, and the spiking threshold differs from the primary dendrite (Figure 6a). Then, we analyzed the signal flow from the tertiary dendrite to the secondary dendrite (Figure 6c), and finally to the soma (Figure 6d). In Figure 6c, the *V_tertiary_*/*V_secondary_* has no noticeable discontinuity, and in general, the relationship is linear, with only a slight deviation from sublinear to supralinear. The continuity occurs because the depolarizations are viewed away from the soma. In our analysis of the membrane potential, we found occurrences of backpropagation and dendritic bursting that are not conveyed into the soma. Then, if we view the signals from the tertiary apical branch to the soma (Figure 6d), the *V_tertiary_*/*V_soma_* relationship is virtually the same as that in Figure 6b. This relationship results from the signal passing through the secondary dendritic branch.

Therefore, even though the signal from a point in the dendritic arborization is viewed from the soma, and there is a distinguishable difference between the output levels in the subthreshold and suprathreshold regions, the thresholding function is still not sigmoidal. Also, the results shown in Figure 6 denote the subunit-independence of each dendritic branch.

### 3.3. Dynamic Nonlinear Thresholding Function

Previously, we indicated that the subthreshold and the suprathreshold regions of individual dendrites with within-branch inputs overlap. We further characterized the dendritic integration curve and discovered that the overlapping regions result from shifting the IO curve attributed to the increase in the number of activated synapses during each simulation. Figure 11 of Supplementary Materials llustrates the clustering of *V_expected_*/*V_observed_* per number of activated synapses, *n*. Furthermore, the correlation coefficient *r* between the *V_observed_* and *n* establishes the influence of synapses in dendritic integration (*r* = 0.43 for primary apical dendrite, *r* = 0.36 for secondary apical dendrite, and *r* = 0.47 for tertiary dendrite).

**Figure 7.**
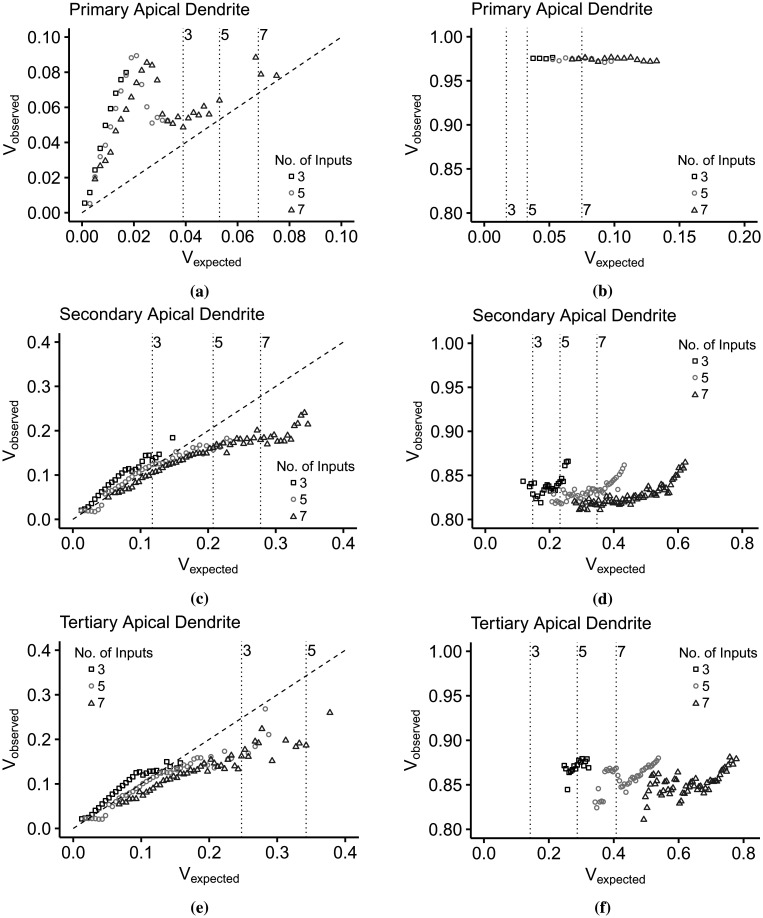
Influence of activated synapses in dendritic integration. In the subthreshold plots, the vertical lines indicate the start of somatic activation per number of inputs, while in the suprathreshold regions, the lines indicate the limit of subthreshold depolarization. In the subthreshold regions (left column), the dendritic integration curve shifts to the right as the number of inputs, *n*, increases. The range of *V_expected_* causing output depolarization in the suprathreshold regions (right column) also shifts to the right with increasing *n*. (a) In the primary branch, the dendritic integration is strongly supralinear, while the integration in (c) the secondary and (e) tertiary branches, the inputs results in dendritic integration shifting from supralinear to linear to sublinear. Then, in (b) the suprathreshold region of the primary apical branch, the *V_expected_* span per *n* are clustered close to each other, while as the branch becomes more distant from the soma, (d) in the secondary, then in the (f) tertiary branch, the span of *V_expected_* becomes more distinct.

We discovered that the overlapping sub- and suprathreshold regions displayed in Figure 5 are indeed not outliers but pertain to the shifting of dendritic integration relative to *n*. We present in Figure 7 the dendritic integration in the subthreshold (left) and suprathreshold (right) regions driven by increasing *n*. The vertical lines in the subthreshold regions indicate the minimum *V_expected_* necessary for a successful somatic spike, while the vertical lines in the suprathreshold regions correspond to the range of *V_expected_* in the subthreshold regions. These limits shift to the right as *n* increases. This behavior demonstrates that the dendritic branch has a dynamic threshold in that the input summation required for somatic spiking varies and is dependent on *n*.

In the primary dendrite (Figure 7a), the dendritic integration is strongly supralinear as it reaches *V_observed_* = 0.09 and then drops down to slightly above the linearity. In the suprathreshold region (Figure 7b), the IO pairs per *n* cluster together and slightly overlap with the neighboring clusters. However, in the secondary (Figure 7c) and the tertiary (Figure 7e) dendrites, we can see the difference in dendritic integration for each *n* in the subthreshold regions. When *n* = 3, the dendritic integration is slightly supralinear (0 < *V_expected_* < 0.1475). For *n* = 5, the integration is linear between [0, 0.1275] and starts to turn into sublinear. Lastly, for *n* = 7, the integration starts as slightly sublinear then moves into strongly sublinear, which plateaus at *V_observed_* ≃ 0.2. For each *n* in the suprathreshold region (Figure 7d), the continuity of dendritic integration, from sub- to suprathreshold, becomes distinct as the minimum *V_expected_* for spiking coincides with the maximum subthreshold *V_expected_* (determined by the vertical lines). The tertiary branch also exhibits the supralinear-linear-sublinear relationship of *V_expected_* and *V_observed_* in Figure 7c, except that the IO curve does not stabilize with a further increase of *V_expected_*. Likewise, in the suprathreshold region, the clustering of IO pairs is more distinct, and there is a clear separation between lower and upper *V_expected_*. Overall, dendritic integration is a nonlinear function displayed in the IO curves in Figure 5. Further analysis of the IO pairs tells us that this nonlinearity consists of multiple linear and nonlinear functions, which can be supralinear, linear, and sublinear, relative to the intensity of the driving force produced by the synapses.

So what do these imply? It tells us that (1) the dendritic branch performs integration independent from the neighboring dendrites, (2) dendritic integration is a dynamic process dependent on the amount of driving force, as well as the number of activated synapses, and (3) the thresholding nonlinearity is the collective effect of linear and nonlinear integration (supralinear-linear-sublinear).

### 3.4. Spatiotemporal Dendritic Abstraction

The dendritic abstraction consists of elements, namely the signal propagation, the linear summation, and the thresholding function. We established beforehand that the voltage decay and time delay functions (Equation 3) in the dendritic abstraction (Figure 3c) characterize the spatiotemporal model attributes of the dendritic branch under consideration. In contrast, the thresholding function is a time-independent and dynamic model based on the amplitude summation and number of incoming input signals, as shown in Figure 5 and Figure 7. We used multiple linear regression analysis to determine the thresholding function in each abstraction, where the model parameters were determined by employing machine learning algorithms performed using the R programming language. The lm and step regression functions provide a direct and efficient way of performing regression analysis. Attached in the supplementary materials is the R code for model prediction.

**Table 4.** Dynamic thresholding function parameters.

Branch	*a*	*b*	*c*	*d*	*θ*	*V_max_*	Multiple R^2^
Primary	-0.023	7.900	0.007	-0.865	0.150	0.975	0.991
Secondary	0.011	1.357	0.004	-0.115	0.200	0.830	0.943
Tertiary	0.017	1.395	-0.001	-0.107	0.270	0.850	0.997

**Figure 8.**
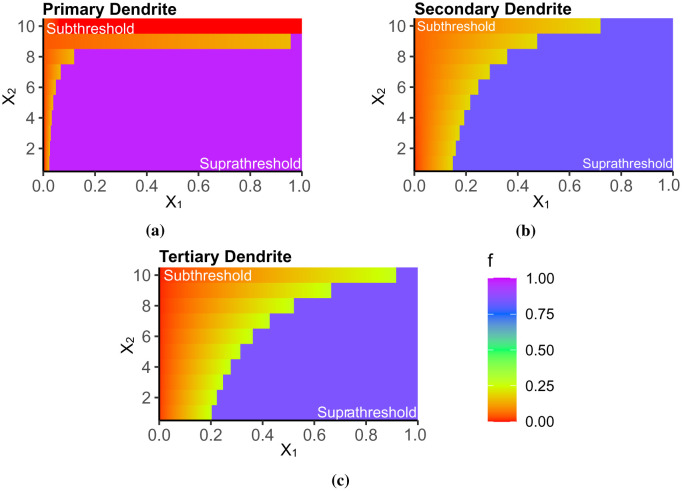
The following are the thresholding functions for each dendritic branch and with respect to the parameters *X*_1_ (input summation) and *X*_2_ (number of inputs from 1 to 10). For all dendrites, the amount of *X*_1_ required to produce an AP increases as the number of activated inputs increases. Thus, in (a), an input level of ~0.02 can result in an AP. Furthermore, as the dendritic branch becomes more distal from the soma, from the (b) secondary to (c) the tertiary dendrite, greater *X*_1_ is required. The proposed thresholding function is, therefore, consistent with the results presented in the previous sections.

The training set comprises 70% of the IO dataset in the subthreshold region, while the remaining 30% was for the testing set. The model which provides the best fit has the form

$$g=a+bX_{1}+cX_{2}+dX_{1}X_{2},$$ (8)

$$f=g\text{ if}g\leq \theta \text{ or }f=V_{max}\text{ otherwise}.$$

where the variables *X*_1_ and *X*_2_ correspond to the summation of inputs arriving at the thresholding point and the number of activated synapses during simulation, respectively. Then, *g* is the subthreshold integration function, while *f* is the overall (sub- and suprathreshold) asymmetric function separated by the spiking threshold *θ*. *V_max_* is the maximum constant depolarization of the dendritic branch. The parameters *a*, *b*, *c*, and *d* were identified using the regression analysis. Refer to Table 4 for the parameter values. The thresholding function, *f*, determines the input intensity that drives the spiking mechanism if the branch is connected to the soma. Refer to Equation 6. In other cases, if the branch under consideration is connected to a proximal branch, *f* determines the normalized signal coming out of the dendritic branch that will be integrated with the other inputs of the proximal branch. Here, *f* is a piecewise function whose subthreshold value is defined by *g*. Then, *g* > *θ* means an AP is generated, forcing *f* to be equal to *V_max_*. This activity is observable in Figure 7. For every number of inputs (*X*_2_), the input summation (*X*_1_) necessary to produce a suprathreshold activity is shifting; thus, we have here a dynamic thresholding function. Figure 8 shows the variation in *f* with increasing *X*_1_ and *X*_2_. The required *X*_1_ needed to cross the suprathreshold region increases with *X*_2_, in agreement with the results in Figure 7. However, in the primary dendrite where *X*_2_ > 8 (Figure 8a), the soma must be forced to spike when *X*_1_ > 0.25 done by changing *f* to *V_max_*, which is due to the large gap between the sub- and suprathreshold regions in the primary-soma connections (Figure 6a). In addition, the variations of *f* for the secondary dendrite (Figure 8b) and the tertiary dendrite (Figure 8c) were comparable with the measured changes in their respective *V_observed_* (Figure 7), where the threshold in terms of the input summation shift from left to right with the increasing number of inputs.

**Figure 9.**
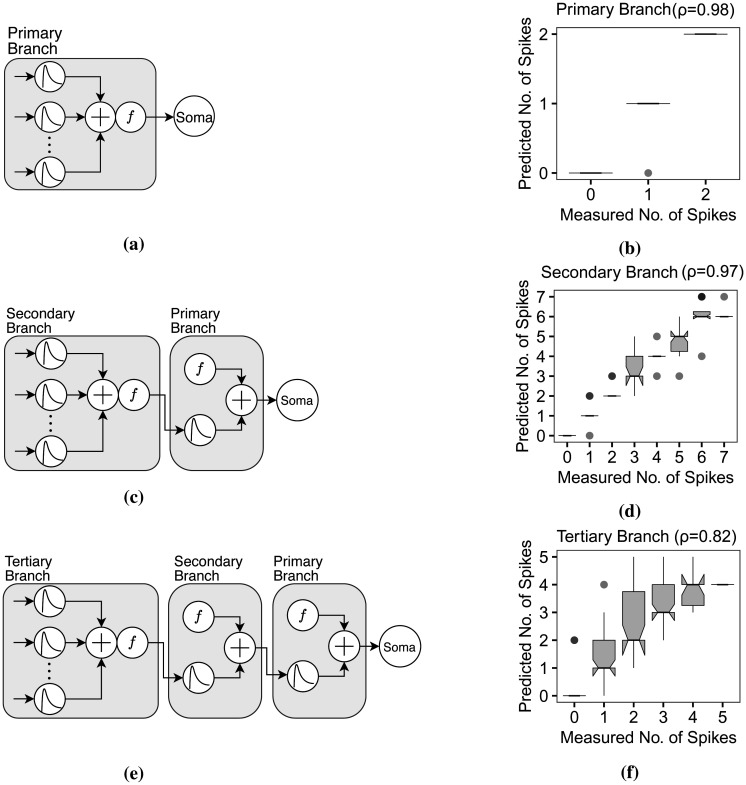
Dendritic abstractions. (a) The dendritic abstraction for the primary dendrites includes the linear summation of synaptic inputs and the thresholding function *f*, whose output directly drives the somatic spiking. (b) The boxplot, a comparison of the measured and expected spiking in the primary dendritic branch within a 200 ms sliding window, indicates a successful predicting capability (*ρ* = 0.98) of the dendritic abstraction in (a). (c) The output of *f_secondary_* does not enter the primary dendrite as input but instead flows individually, with delay and decay, through the primary branch for linear summation with *f_primary_* output, before reaching the soma. (d) The boxplot where *ρ* = 0.97 suggests that the dendritic abstraction in (c) effectively predicts the somatic spiking. It indicates that spiking is more frequent on the secondary than in the primary dendrites. (e) The tertiary branch illustrates a dendritic abstraction similar to (c) in that the output of *f_tertiary_* flows individually through the secondary branch for linear summation with the output of *f_secondary_* until it reaches the soma. (f) The measured and expected somatic spikes comparison validates the dendritic abstraction (*ρ* = 0.82).

In the Supplementary Materials - Figure 12, superimposed on the measured depolarization is the predicted *V_observed_* points. The plots show that the proposed thresholding function indeed follows the dynamic characteristics of dendritic integration. The dendritic integration shifts to the right and varies from supralinear to linear and sublinear described in Figure 7. The resulting multiple R^2^ (Table 4), which are above 0.9, validate the predictions. In addition, Figure 13 in the Supplementary Materials shows the predicted depolarizations in the basal subtree.

We simulated the biological CA3 pyramidal neuron model by activating random synapses in each dendritic branch for 5000 ms (10 simulations each branch). With the same synaptic inputs measured from the dendritic spine heads, we implemented the proposed dendritic abstraction in Figure 3c. We predicted the dendritic dynamics and somatic spiking using the corresponding propagation model (Equation 3) and the dynamic thresholding function (Equation 8). For instance, Figure 14 in the Supplementary Materials shows the raster plot of random synaptic inputs along the primary apical dendrite and the corresponding observed and predicted somatic spiking. Then, we compared the measured with the predicted spiking activity within a 200 ms sliding window.

Figure 9a shows the dendritic abstraction of the primary apical dendritic branch (simulated with *n* ∈ [4, 10] and *spike interval* ∈ [38, 67] ms). The output of the thresholding function is the direct input to the soma. The number of measured and predicted spikes are from 0 to 2 for every 200 ms sliding window. The boxplot in Figure 9b shows that the dendritic abstraction successfully predicted the somatic spiking where the R^2^ *ρ* is 0.98. Figure 9c shows the dendritic abstraction for the secondary dendritic branch (*n* ∈ [2, 10] and *spike interval* ∈ [40, 71] ms). In this case, the output from the secondary thresholding point independently flows through the primary branch with considerable delay and decay before reaching the soma. Here, the primary dendrite is, therefore, equivalent to a multiplexer cable, or a waiveguide [58]. The linear relationship between the means in the corresponding boxplot (Figure 9d) indicates a good prediction capability of the dendritic abstraction (*ρ* = 0.97). The tertiary branch exhibits independence from its mother dendrites and signals multiplexing (Figure 9e) (*n* ∈ [3, 10] and *spike interval* ∈ [36, 68] ms). Given the linear relationship of measured and predicted spikes in Figure 9f, and that *ρ* = 0.82, suggests the dendritic abstraction describes the spiking activity of the biological neuron model.

## 4. Discussion

### 4.1. Opting for a natural neuronal response in IO quantification

Biological neuron models based on experimental evidence provide a direct means of manipulating neuronal characteristics, such as ionic channel distribution, biophysics, morphology, and synaptic inputs, that are challenging to control *in vitro* or *in vivo* [11]. We created a morphologically-realistic and biologically-based CA3 pyramidal neuron model (Figure 1a) and studied the dendritic integration of individual branches via analyzing their transfer functions or IO relationships. The goal here is to find the corresponding thresholding function, specifically, to identify the instance that the dendritic integration curve crosses the suprathreshold from the subthreshold region. The first step is to determine the approach for the IO quantification process. The shape of the IO relationship varies, dependent on (1) the quantification process: single-pulse or paired-pulse stimulation protocol, and blocked spiking mechanisms, and (2) the parameters under consideration: linear for passive mechanisms, nonlinear for the active mechanism (Na^+^ channels and NMDA receptors), supralinear to sublinear for increasing driving force. These quantification procedures limit the dynamic response of the neuron and, consequently, the dendritic integration process. Instead of concentrating on one parameter and constricting the others, we proposed an IO quantification process that opted for natural dendritic response by letting the soma spike spontaneously without blocking the spiking mechanisms to conserve the full range of dendritic activity. However, this process poses some challenges. When the soma generates APs, back-propagating signals move swiftly from the soma through the dendritic tree [23], causing consecutive spikes in the thresholding point. Clustered and strongly-activated synapses create regenerative Na^+^ spikes localized within the branch as well [33]. Additionally, voltage-gated Ca^2+^ spikes amplify local Na^+^ currents causing somatic bursts firing in CA3 neurons [59]. During simultaneous synaptic and somatic activities, what causes the dendritic spikes is unclear; it is either the dendritic activity or the backpropagation [60]. These dendritic spikes overlap with the peak depolarization of successful spikes (spikes that cause AP generation). Therefore, we removed the IO points caused by dendritic spikes in the dataset, those points whose observed depolarization are between the threshold and 0.8. With this process, we still conserved the spontaneous dynamics of the dendrites and examined the full range of dendritic integration, from sub- to suprathreshold activities.

### 4.2. Branch-specific dendritic integration implements a dynamic thresholding function

Implementing the proposed IO quantification method, the IO curves in Figure 5 and Figure 7 describe a dendritic integration with the following features. First, dendritic integration is dynamic. Integration in the primary apical and basal dendrites are highly supralinear (Figure 5a), while distal dendrites exhibit a nonlinear integration varying from supralinear to slightly sublinear (Figure 5c and 5e). Two-photon imaging and glutamate uncaging on CA3 pyramidal neurons indicate that the apical and basal proximal dendrites perform highly supralinear integration, mainly influenced by NMDA receptors [24]. Localized regenerative events during activation of voltage-gated channels contribute to the nonlinearity in distal branches [11]. IO curves seen as a whole depict a single nonlinearity described by Poirazi et al [17]. The IO curve starts linearly for weak signals, then progresses to supralinear for intermediate signals. As the curve intersects the line separating the supra- and sublinear region, the IO curve becomes sublinear and plateaus as the input continues to increase until the input is sufficient for somatic spiking. Further analysis of the IO curves showed that a single branch could change its integration mode, between supralinear, linear, and sublinear, as shown in Figure 7.

Second, the spiking threshold varies with the number of activated synapses. Using whole-cell recordings in the CA3 region of cultured rat hippocampus, Soldado et al. [61] discovered that the transition from the subthreshold to the suprathreshold (during firing activity) is not a static characteristic of the neuron. Our analysis determined that the number of activated synapses, not only the input summation in general, has considerable influence on the dynamic dendritic behavior. The threshold or the amount of synaptic inputs required to generate an AP is dynamic [62], and varying synaptic input patterns influence the form of dendritic depolarization [63]. *In vivo*, the threshold varies with the number of inputs and spiking history [33], [62]. Figure 7 also illustrates the shifting from left to right of the firing threshold. The minimum amount of input summation necessary to cause a successful spike increases as the number of active synapses increases. In the primary dendrite (Figure 7a), the linear part of the integration mode (0 ≤ *V_expected_* ≤ 0.02) shifts by changes in slope. In the secondary (Figure 7c) and the tertiary (Figure 7e) branches, the integration mode is slightly supralinear at *n* = 3, linear at *n* = 5, and sublinear at *n* = 7.

Lastly, dendritic integration is branch-specific. Dendritic branches process information independent of the whole neuron, which is evident in the differences in the IO curves of each branch (Figure 7). The amount of driving force required for somatic spiking differs, which is minimal in the primary dendrites and maximal in tertiary dendrites. Branch specificity is also apparent in the varying spiking threshold *θ*, and maximum depolarization *V_max_* developed in the branches (Table 4). The location of the dendritic branch plays a significant role in branch specificity. In Figure 6, we compared the dendritic spiking activity in each branch with the somatic activity. The depolarization at the thresholding point in the apical trunk in relation to the soma is linear, with a large discontinuity between the sub- and suprathreshold (Figure 6a). The relationship between the peak depolarizations in the tertiary and secondary thresholding points is also linear. Contrastingly, the linearity is continuous (Figure 6d). What causes the discontinuity in the apical trunk? When the input summation reaches the threshold, the somatic depolarization instantly generates an AP and peaks to ~40 mV. Dendritic spikes and backpropagation in the non-primary dendrites create peak depolarization between the threshold and AP peak amplitude. Regenerative spikes are localized in a specific branch [23], [33]. As shown in Figure 9b, 9d and 9f, dendritic spiking is more frequent in distal dendrites, ten times more than the soma [33], due to higher input resistance [64]. Depolarization in the secondary (Figure 6b) and tertiary (Figure 6d) dendrites compared with the somatic depolarization display a strongly sublinear relationship in the subthreshold area and constant peak amplitude in the suprathreshold area. Due to compartmentalization, the current flowing to the next compartment decreases, and the signal attenuates [21]. Usual IO transformation methods compare the input summation with somatic spiking. On the other hand, dendritic integration varies depending on the location where the input and the output are measured. Therefore, branch-specific processing of diverse synaptic inputs results in cell-specific activities, as depicted by the experimental recordings from CA1 neurons in the rat hippocampus [65].

This study focuses on the dendritic integration of excitatory AMPA and NMDA synaptic inputs. However, this modeling method does not nullify the branch-specific effect inhibition as inhibitory signals also have properties that shape the IO curve. Interneurons trigger *γ*-aminobutyric acid (GABA) receptors predominantly located along the dendritic shaft near the soma and activate inhibitory signals. Inhibitory signals modulate NMDA signals [66] due to the voltage dependency of the receptors. In addition, local inhibition produces shunting effects [48] that scale down the input summation amplitude and could produce a negative segment on the IO curve. The subtractive property of inhibitory signals shifts the IO relationship to the left [33], produces sublinear summation [67], and selects information for routing [7].

We formulated a dynamic thresholding function (Equation 8) capable of replicating the above dendritic integration features. This piecewise function is a multiple linear regression dependent on both the summation of synaptic inputs and the number of activated synapses. This function is time-independent as most thresholding functions are. Additionally, the function can shift between integration modes, from supralinear to linear and sublinear, while sustaining the overall dendritic nonlinearity. The dynamic thresholding function enhances the computational capacity of the dendritic branch, compared with the commonly used static nonlinearity in GLMs and McCulloch-Pitts neurons. Dynamic threshold, dynamic integration mode (deviating between linear and nonlinear), and input location specificity enhance the computational power of the dendrite as it allows the dendrite to shift from one integration mode to another [11], [68]. These capabilities reveal that a single neuron performs more complicated functions previously associated only with neuronal networks.

### 4.3. Dendritic abstraction with dynamic thresholding function

As mentioned earlier, the thresholding function is time-independent, and the input summation can occur anywhere along the dendritic arborization. Input summation was set on the proximal end of the dendritic branch, a point near the bifurcation, to cover the whole dendritic branch length. We proposed a dendritic abstraction (Figure 3c) that models the spatiotemporal changes of synaptic inputs during propagation. Synaptic inputs are subjected to voltage decay and delay attributed to the location of the input from the thresholding point and the active mechanism along the dendrites (Equation 3). The linear summation of propagated inputs occurs once the inputs reach the thresholding point, followed by the transformation employing the thresholding function.

We demonstrated that the dendritic abstraction models the integration process of the dendritic branch and then proceed with the information transfer scheme from the tertiary branch straight to the soma (Figure 9). In most generalized linear models, the output of one secondary subunit combines with the inputs of the subsequent subunit, the primary branch. However, with this scheme, the neuron does not correctly predict the expected output train. For example, if the output of the secondary branch is equal to 0.18, which is in the subthreshold region, enters the thresholding unit of subunit A, the final output will be a successful spike being that the threshold of A is only 0.15 (refer to Table 4). The difference between the thresholding levels of the two subunits indicates that dendritic subunits perform independent dendritic integration processes and the dendritic length multiplexes branch-specific information. Experiments on neuronal cultures suggest that dendritic arborization forms multiple layers of dendritic integration and independent functional subunits within a layer [23], [25], [69], comparable with multiplex communication in cultured neural networks [70].

An additional component in the dendritic abstraction is the linear summation after the thresholding unit for mother branches (Figure 9c and 9e). This component feeds the simultaneous inputs from different branches into a single spike train. The dendritic tree can, therefore, multiplex multiple information from independent sources [7], [16]. Local dendritic integration and signal multiplexing continue until the information reaches the soma. The soma performs a global integration summing the inputs from the proximal apical and basal dendrites. Sakuma et al. [71] also suggested that the synaptic delays and refractory periods improve and stabilize multiplex communications in neurons. This particular scheme also increases the computational capacity of the neuron. It performs spatiotemporal filtering by confining some information within the specific dendrite. Distal branches produce and confine dendritic spike bursts that do not reach the soma. It also performs information selection. During somatic spiking, the soma blocks inputs, and during this time, some dendritic spikes are unable to reach the soma [7]. Only certain local information from a distal dendritic branch is delivered to the soma. With this scheme, where inputs are independently processed, the neuron can also determine the source of the inputs responsible for the somatic firing.

### 4.4. Dendritic learning scheme

One of the primary mechanisms for synaptic plasticity is strengthening the synaptic connectivity during backpropagation; the synaptic conductances are updated after a successful spike from the soma. This concept of spike-timing dependent plasticity (STDP) means that the learning scheme is global, strengthening the synapses that the backpropagating signal can reach. However, Sardi et al. [1] proposed a paradigm shift in learning, that is, by dendritic learning instead of the slow synaptic plasticity. Recent experimental studies in CA3 pyramidal neurons prove that a single dendritic branch induces branch-constrained synaptic plasticity through NMDA spikes and Ca^2+^ transients [72]. A compartmental model shows that neuromodulator acetylcholine inhibiting K^+^ channels could generate NMDA spiking, which is necessary for facilitating local plasticity [73]. The dendritic abstraction we proposed divides the neuron into independent computational subunits, which allows synaptic plasticity to occur faster within the compartment. Because each subunit performs individual signal summation and transformation, it can keep track of its dendritic activity. Then if *f* produces a value above the threshold, it signals the dendritic branch to perform compartmentalized synaptic plasticity, strengthening its synapses without waiting for the backpropagating signal to arrive. CA3 neurons can, therefore, perform local plasticity via NMDA-spike dependent long-term potentiation in addition to soma-dependent STDP [74], [75].

## 5. Conclusion

Overall, we developed an IO transformation process and modeled the corresponding branch-specific integration. The thresholding function describes a dynamic integration process. We also formulated a dendritic abstraction incorporating the spatiotemporal characteristics of synaptic inputs while traveling down the dendritic length. We suggest further investigation of dendritic integration by merging both experimental and computational studies. Current physiological experiments are still limited in spatiotemporal resolution. Besides, we suggest examining a pyramidal neuron with a more complex dendritic arborization and developing the dendritic abstraction with excitatory and inhibitory inputs and branch-specific learning rule, although complicated arborization equates to exhausting manipulation. It has been suggested that neurons perform more complex computations comparable with neuronal networks. Therefore, further investigation into dendritic processes helps understand neuronal functions employed in biomedical, artificial intelligence, and neuromorphic applications [76], [77].

## Supplementary Materials

**Figure 10.**
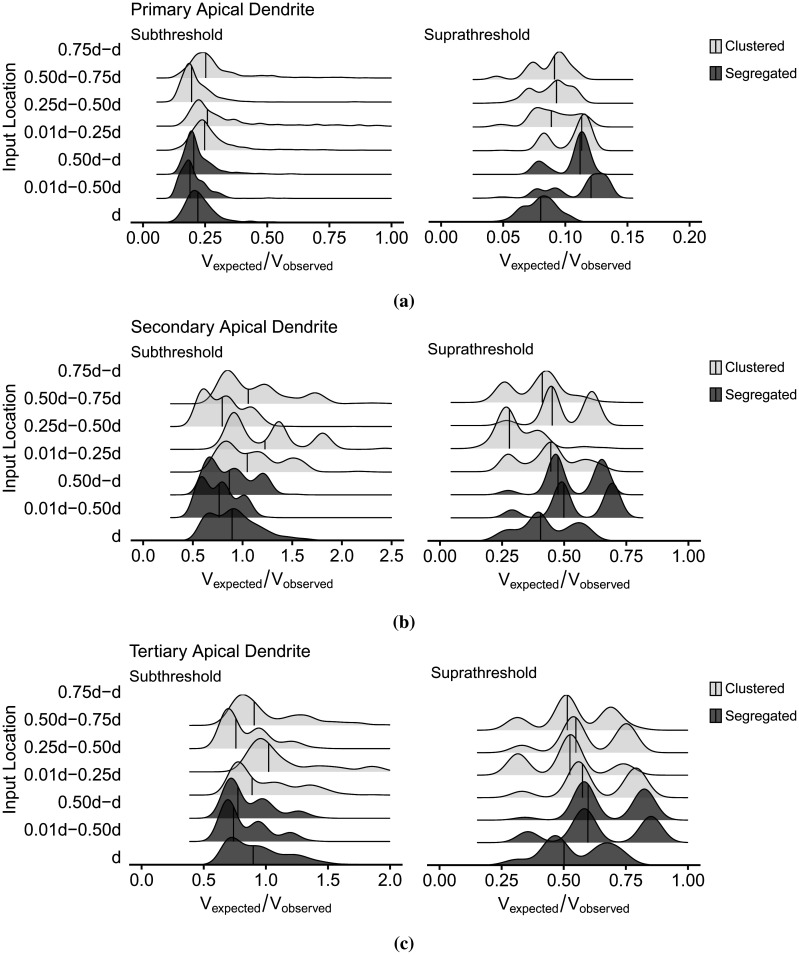
Density plots of segregated and clustered within-branch synaptic inputs located along the dendritic length *d*. The results indicate no considerable correlation with the dendritic depolarization and location of activated synapses since the spatiotemporal characteristics of the inputs and dendrites were already expressed in the propagation models.

**Figure 11.**
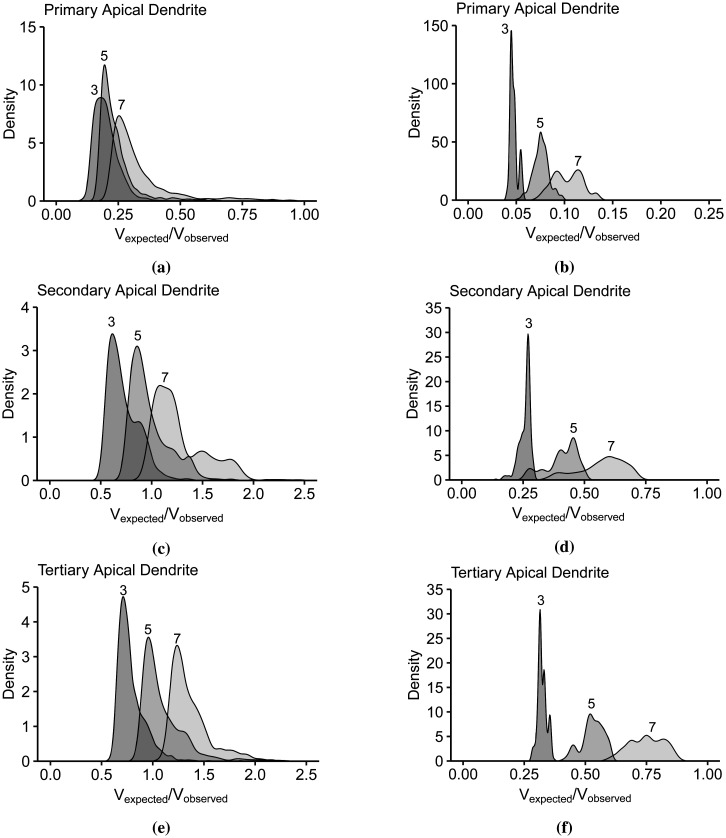
The density plots of input summation *V_expected_* show the clustering of thresholding point depolarizations *V_observed_* per number of synaptic inputs (3, 5, and 7).

**Figure 12.**
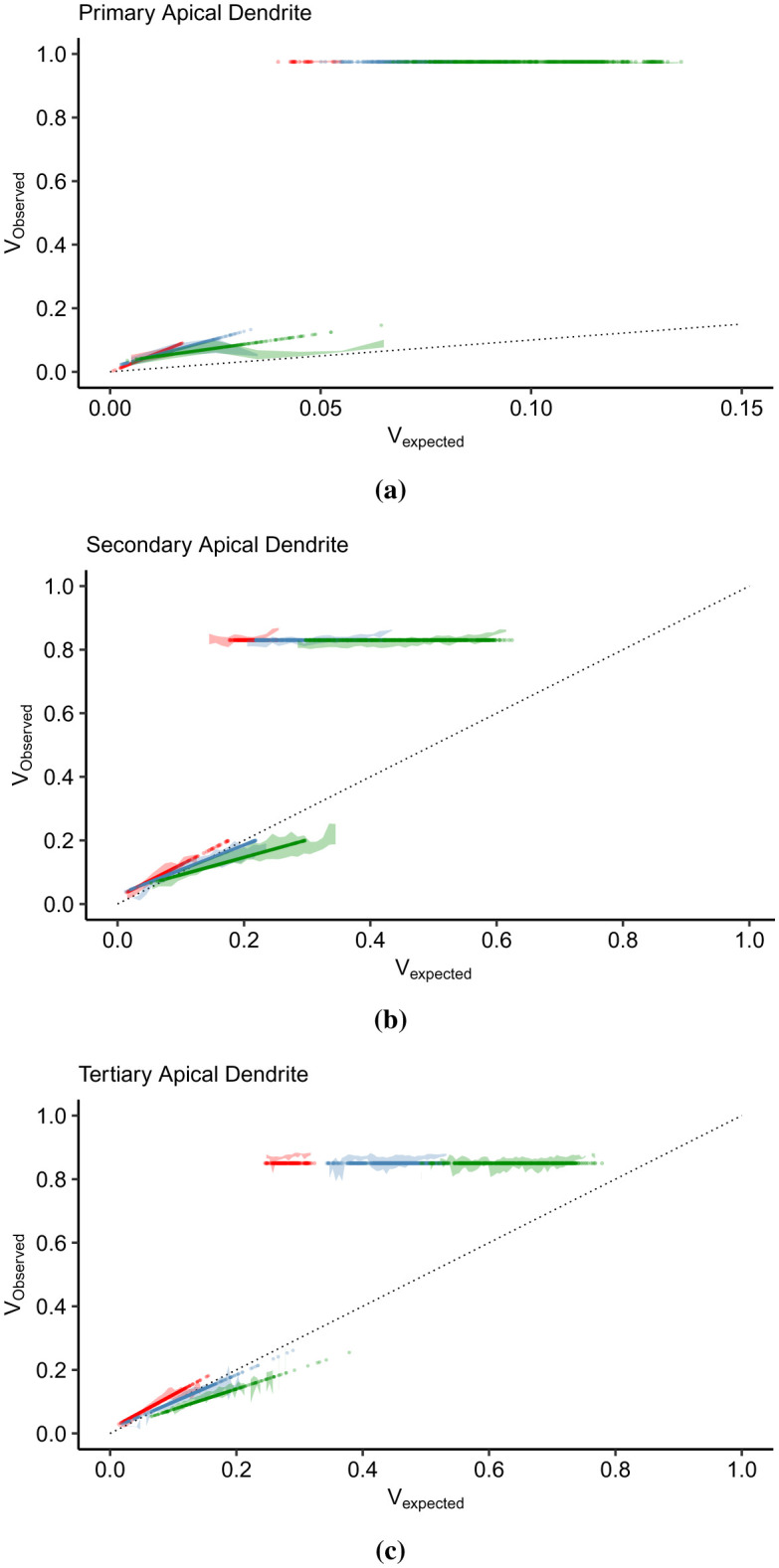
Predictions using the dynamic thresholding function. The predicted *V_observed_* (points) superimposed on the measured *V_observed_* at the thresholding point in the (a) primary, (b) secondary, and (c) tertiary apical branches. The prediction follows the behavior of the observed depolarization, wherein the dendritic integration shifts to the right with an increasing number of activated synapses, *n* = 3 (red), *n* = 5 (blue), and *n* = 7 (green).

**Figure 13.**
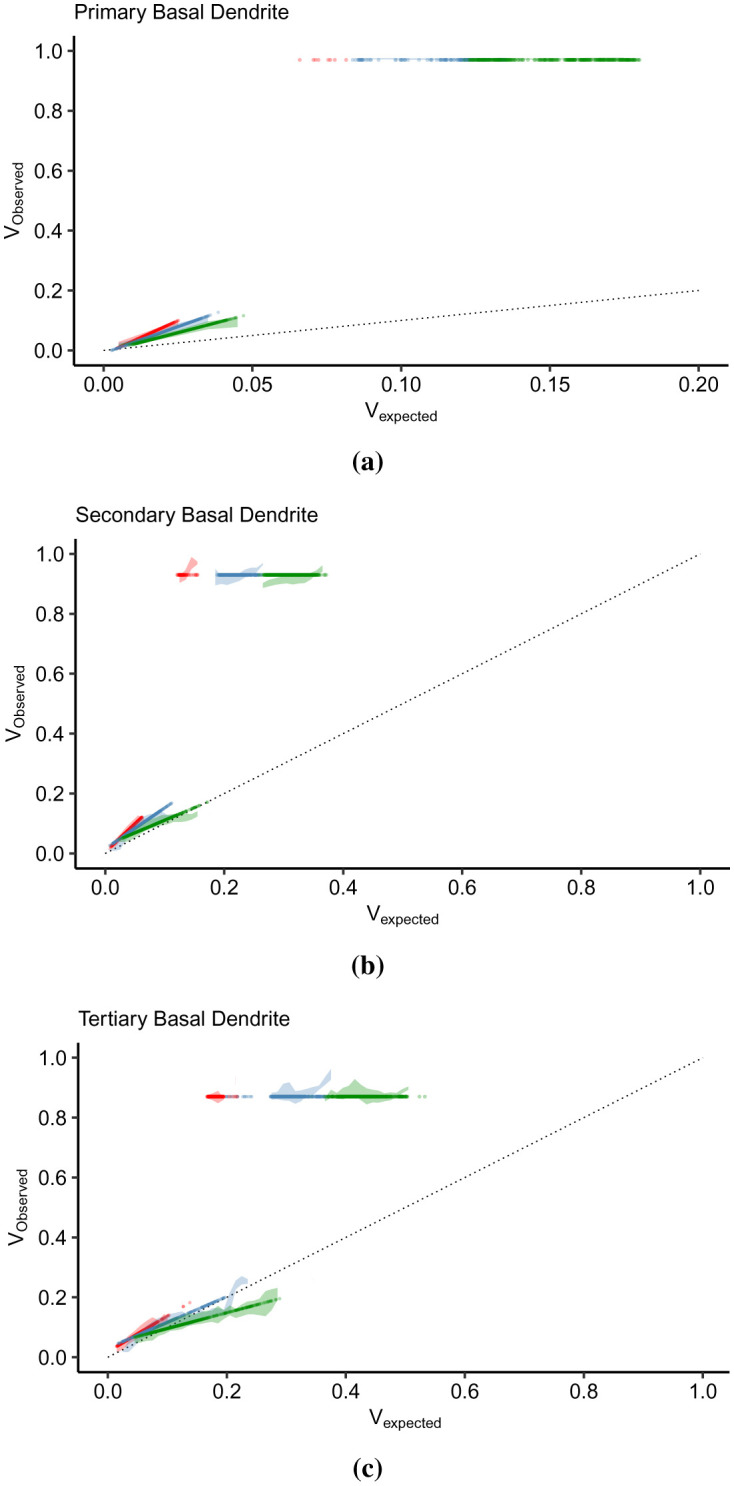
Predictions in the basal dendrites with an increasing number of activated synapses, *n* = 3 (red), *n* = 5 (blue), and *n* = 7 (green). The basal dendrites (the unitary dendritic subtree) show a similar dynamic nonlinearity as in the apical dendrites. The thresholding functions have the following parameters: (a) *a* = −0.0189, *b* = 5.9470, *c* = 0.0019, *d* = −0.4808, *V_max_* = 0.97, *R*^2^ = 0.934, (b) *a* = −0.0118, *b* = 2.6687, *c* = 0.0055, *d* = −0.2611, *V_max_* = 0.93, *R*^2^ = 0.7223, and (c) *a* = 0.0003, *b* = 1.6828, *c* = 0.0061, *d* = −0.1650, *V_max_* = 0.87, *R*^2^ = 0.661.

**Figure 14.**
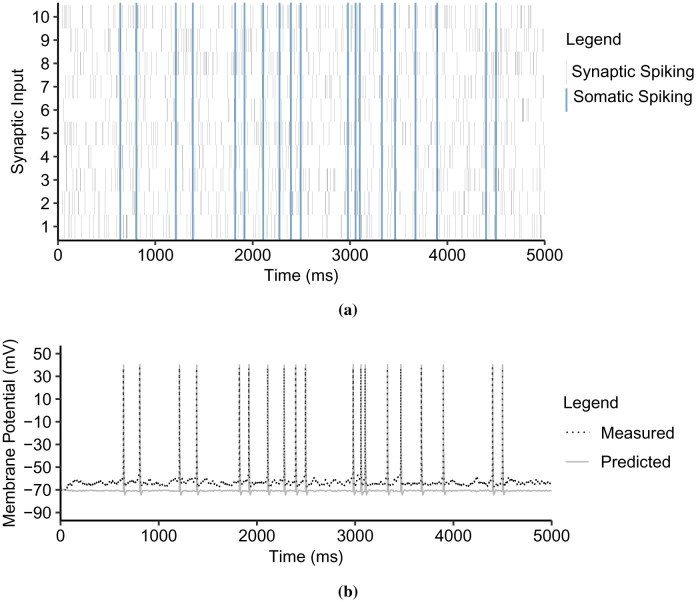
(a) Sample random synaptic spiking activity in the primary apical dendrite and the corresponding somatic spiking. (b) The measured somatic spiking was reproduced using a Hodgkin-Huxley mechanism.
